# Influence of the protocol of fibroin extraction on the antibiotic activities of the constructed composites

**DOI:** 10.1007/s40204-015-0039-x

**Published:** 2015-07-04

**Authors:** Wafa I. Abdel-Fattah, Nagwa Atwa, Ghareib W. Ali

**Affiliations:** 1 Biomaterials Department, National Research Centre, Tahrir St. Dokki, Cairo, Egypt; 2 Chemistry of Natural and Microbial Products Department, National Research Centre, Tahrir St. Dokki, Cairo, Egypt

**Keywords:** Silk fibroin, Nanosilver, FTIR-deconvolution, Bactericidal activity, Band gap

## Abstract

The effect of the solvents for silk fibroin (SF) extraction on its antimicrobial activity was studied. Extraction protocols were performed using LiBr (SF_L_) and Ajisawa’s reagent (CaCl_2_:ethanol:H_2_O) (SF_C_). The morphological and structural characteristics of the extracted SF and their composites were assessed. Corresponding bactericidal activities against *Staphylococcus aureus* (ATCC 25923), *Escherichia coli* (ATCC 25922) and *Pseudomonas aeroginosa* (ATCC 27853) were performed. The resulting solutions were either casted into films or individually incorporated into composites of silver nanoparticles (NS) embedded into chitosan fragments (Cs) through γ-irradiation. Films of SF, obtained by using the two solvents, as well as the final prepared composites of SF, NS and Cs were analyzed using XRD, FTIR, SEM, TEM and zeta potential at several pH values. The band gap values were calculated. The results proved that, although SF_C_ consumed shorter gelation time, yet SF_L_ exerted higher antibiotic activity against the tested microorganisms. Moreover, the final composites had the ability to significantly reduce the growth of these medically relevant bacteria and are, therefore, recommended as a novel natural antibacterial biomaterial for several biomedical applications.

## Introduction

Biomaterials have been increasingly applied to improve surgical procedures of missed bones to restore the quality of life. Several millions of patients worldwide need bone grafts and other orthopedic as well as dental surgeries. The demand for biomaterials increases every year. It is crucial that these devices should be non-toxic, biocompatible and non-immunogenic.

Silk has been used for thousands of years in the textile and for about a century for suture materials. Recently, silk is reported as one of the most chosen candidates for biomedical applications since it exhibits biodegradability and environmental stability with excellent mechanical properties (Altman et al. 2003).

The domesticated silkworm, *Bombyx mori*, spins silk with distinctive properties. An insoluble protein, silk fibroin and glue-like protein, sericin, combined to form the silk. It is composed of three components: heavy (H) chain fibroin (350 kDa), light (L) chain fibroin (25 kDa) and P-25 protein in a molar ratio of 6:6:1, respectively. Structural analysis of the H-chain possesses two types of molecular conformations being α- and β-helix. The former is non-crystalline and soluble in water while the later is highly stable organized and insoluble in water (Motta et al. 2002). Silk I is mainly formed of α-helix sheets, while that containing a higher percentage of β-helix is termed silk II (Inoue et al. 2000).

Silk fibroin is an approved FDA product for many applications such as surgical sutures (Rujiravanit et al. 2003), drug delivery components and tissue engineering (Sofia et al. 2001) beside burn wound dressings (Santin et al. 1999), enzyme immobilization matrices (Acharya et al. 2008), vascular prostheses and structural implants (Dalpra et al. 2005; Meinel et al. 2005). However, some concerns have been raised regarding the use of silk as a medical biomaterial since several adverse immune responses, caused by sericin as well as the wax-like material found on the fibers, were reported (Nagarkar 2010). However, pure silk fibroin constructs are reported to be biocompatible, biodegradable with minimal inflammatory reactions (Altman et al. 2003). Such pure silk fibroin is obtained by the degumming of silk fibers where the aqueous solution of the protein is converted into several biomaterial forms such as hydrogels, films, sponges and non-woven mats (Vepari and Kaplan 2007).

Silk fibroin hydrogel is reported to have considerable attention for drug delivery and tissue engineering applications (Hanawa et al. 1995; Fini et al. 2005). The gel strength and gelation rate were reported to be greatly dependent on the pH and solution concentration. When the electrostatic repulsion between the macromolecules is sufficiently reduced at the isoelectric point of silk fibroin (pH 3.8–3.9) gelation occurs (Haider et al. 1992). Interestingly, the low-pH-induced silk fibroin hydrogel proved to impart better healing results when used as bone filling biomaterial (Fini et al. 2005).

A true solution of SF protein is very difficult to obtain due to its high molecular weight (500–750,000 Da) and its crystallinity, caused by the intermolecular hydrogen bonds, as well as its hydrophobic nature (Simmons et al. 2006). Various techniques were proposed to dissolve silk fibroin. These involved the use of harsh, chaotic solvents such as strong acids or ionic concentrated salt solutions. Scientists used concentrated sulphuric, hydrochloric or nitric acids to dissolve the fibers (Sonthisombat and Peter 2004). Moreover, different ionic liquids including LiBr (Chen et al. 2001), CaCl_2_ or Ajisawa’s reagent (Liang and Hirabayashi 1992), calcium nitrate in methanol (Mathur et al. 1997), aqueous lithium bromide and ethanol (Matsumoto and Uejima 1997; Matsumoto et al. 1997), aqueous lithium thiocyanate (Agarwal et al. 1997) and aqueous sodium thiocyanate (Sun et al. 1997) were reported.

The aim of the present work was to study the dissolution of silk fibroin, from *Bombyx mori*, using either LiBr solution or Ajisawa’s reagent as models of ionic liquids. The obtained solutions were casted into films and their antibacterial activities, against medically relevant gram positive and negative bacteria were compared. The silk fibroin obtained by either protocol was incorporated into a nanosilver/chitosan composite (Ag6d3), previously synthesized and assessed by our team (Abdel-Fattah et al. 2014). The two solvents were selected as they feature the most common protocols applied in silk fibroin extraction. Salvador et al. (2013) studied the effect of extraction protocol on the chemical and mechanical properties of the obtained silk fibroin. However, studies on the influence of extraction protocol on silk fibroin bactericidal activity are rare. The present work is concerned with tackling this deficiency. According to the future biomedical demand, the extraction protocol could be selected as extraction by the Ajisawa’s reagent will need shorter gelation time. On the other hand, extraction protocol with LiBr will result in more efficient bactericidal functions.

## Materials and methods

### Materials

Silk fibroin was extracted from Egyptian *Bombyx mori* (Silkworm). Purchased chemicals were sodium carbonate (Fine-Chem. LTD), lithium bromide (M.wt., 86.845, 99 %, Molychem.), calcium chloride (M.wt. 110.99 g, 98 %, ADWIC), chitosan (85 % deacetylated, Oxford chemical Co), silver nitrate (M.wt. 169.87 g, 99.8 %, Fine-Chem. LTD), glutaraldehyde (50 %, ADWIC), acetic acid and isopropanol (analytical grade). All solutions were prepared in bidistilled water. Dialysis membrane, MWCO 3500 kDa (Pierce, Rockford, IL, USA) was used.

The antibacterial activities of the developed films and composites were tested against *Staphylococcus aureus* (ATCC 25923), *Escherichia coli* (ATCC 25922) and *Pseudomonas aeroginosa* (ATCC 27853) purchased from American Type Culture Collection (ATCC, Manassas, VA). Strains were cultivated in Mueller–Hinton (MH) broth (Guangdong Huankai Microbial Science and Technology Co Ltd, Guangzhou, China).

### Methods

#### Silk fibroin extraction

*Silk fibroin degumming process* Egyptian *Bombyx mori* cocoons weighing 20 g (pupae and inner envelope removed) were degummed to remove the sericin. Degumming was performed by boiling for 3 consecutive times, 1 h each, in an aqueous solution of Na_2_CO_3_ (1 g/L). Degummed silk was then washed several times with bidistilled water and oven dried at 40 °C (Salvador et al. 2013). Continuous boiling was avoided to prevent sericin accumulation in the degumming solution.

**Fig. 1:**
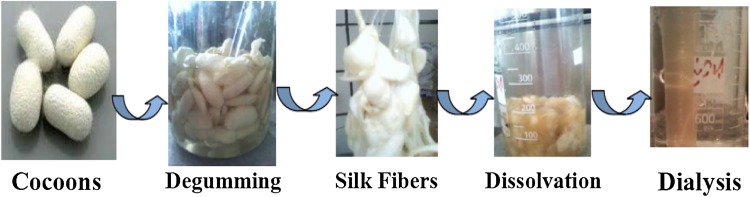
Silk fibroin (SF) degumming and dialysis processes for obtaining SF solutions

*Silk fibroin dissolution* The extraction routes using either LiBr solution (9.3 M) or Ajisawa’s reagent composed of CaCl_2_:C_2_H_5_OH:H_2_O in 1:2:8 molar ratios were compared in concentration of 10 %W/V and designated as SF_L_ and SF_C_, relevant to LiBr and Ajisawa’s reagent, respectively (Salvador et al. 2013). Both batches were subjected to continuous stirring at 60 °C until complete dissolution of the fibers followed by filtration. LiBr and CaCl_2_ remnants were removed by dialysis against bidistilled water for three consecutive days at room temperature using a cellulose membrane (Fig. 1).

#### Films processing

Silk fibroin/nanosilver/chitosan composites (SFNSCs) were manipulated using SF extracted via both regimes by individually adding 3.5 ml of each prepared silk fibroin solution to 1.5 ml of Ag6d3 solution. The later was previously prepared by subjecting an aqueous solution of 0.6 g silver nitrate and 3 g chitosan to the selected γ ray irradiation dose of 75 kGy (Abdel-Fattah et al. 2014; Chen et al. 2007). Glutaraldehyde was added to each solution (0.2 % V/V), mixed via magnetic stirring for 2–3 min, casted on polyethylene plates and then dried at room temperature. The obtained composites were designated as SF_L_NSCs and SF_C_NSCs corresponding to LiBr and Ajisawa’s reagent, respectively.

#### Samples characterization

UV–Vis spectra of the solutions were obtained using Junway 6100 spectrophotometer to measure absorbance over a range of 200–1000 nm semi-quantitatively.

The X-ray powder diffraction (XRD) analysis was performed with Philips X’pert Pro X-ray powder diffractometer using Cu Kα radiation (*λ* = 1.5418 Å) at a scanning speed of 0.3 S. The applied voltage and current were 40 kV and 40 mA, respectively.

Fourier transform infrared (FTIR) spectra were recorded using JASCO 430 FTIR (Japan) spectrometer equipped with TGS detector. The samples were prepared by compressing their powder with KBr (2/198 mg). The spectra were taken at 4.0 cm^−1^ resolution. 64 scans were accumulated to get a reasonable signal to noise ratio.

Transmission electron microscope (TEM) images were obtained (JEOL-1230) at an accelerating voltage of 100 kV. The samples were completely dispersed using an ultrasonic homogenizer for 6 min, and then one drop was placed on a carbon-coated copper grid. Samples were left to dry at ambient temperature.

Zeta potential values were measured using the laser zeta meter (Malvern zeta seizer). A silver chitosan nanoparticles aqueous solution (10 % W/V) including NaCl as a suspending electrolyte solution (2 × 10^−2^ M) was prepared. The pH was adjusted to the required values (HANNA pH meter 211). The samples were shaken for 30 min, the equilibrium pH was recorded and the zeta potential measured. An average of three separate measurements was obtained.

#### Antimicrobial activity testing

Bacterial cell suspensions were prepared, for each tested bacterial culture, using sterile normal saline solution (0.9 % w/v NaOH) to obtain a final concentration of 10^7^ CFU/ml by comparison with a 0.5 Mc Farland turbidity standard. Equal weights of each film were individually inserted in test tubes, each containing 10 ml of sterile Mueller–Hinton (MH) broth (composed of g/l: beef extract, 2.0; casein hydrolysate, 17.5 and starch 1.5; pH 7.3 ± 0.2). The medium, supplemented with the samples, was sterilized by autoclaving for 20 min at 120 °C and 1.5 atmospheric pressure. After sterilization, each test tube was inoculated with 100 µl of one of the previously prepared bacterial suspensions and then incubated under moderate shaking of 100 rpm at 35 °C for 24 h (treated microorganisms). Controlled test tubes, containing the same volume of MH medium free of composite films, were inoculated as well using the same inoculum size of the tested strains (untreated microorganisms). The cell growth of the tested bacteria was determined at the end of the incubation period, based on the optical density measurements at a wavelength of 620 nm (CE 595 double beam spectrophotometer. Cecil instrument). Results were expressed in terms of their cell dry weight (CDW) using the relation between the optical density of the cell and their cell CDW (Abdel-Fattah et al. 2014).

#### Morphology of films bacterial cultures

The film specimens were rinsed twice with phosphate buffer solution (pH 7.0) to remove culture media residues and the samples fixation was performed as previously reported (Abdel-Fattah et al. 2014). Finally, the specimens were mounted on copper stubs with double-sided adhesive tape, coated with gold (S150A Sputter coated Edwards-England) and then scanned by SEM (JXA-840A Electron probe micro analyzer-Joel-Japan).

## Results

### Aqueous solubility of silk fibroin

The results showed that LiBr was able to completely dissolve the silk fibroin fibers in a relatively short time (2 h) while the Ajisawa’s reagent needed double incubation time (4 h). After dialysis, the solutions were filtered. The total solid protein concentration of 4 % (W/V) was achieved through drying a known volume of silk fibroin solution and weighting.

### Silk fibroin gelation

**Fig. 2:**
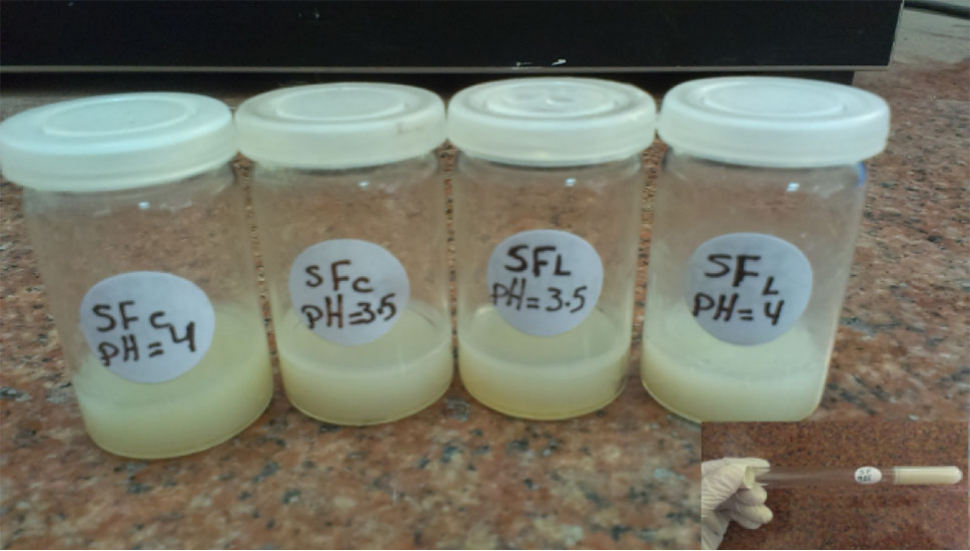
Silk fibroin hydrogels at several pH values. The *inset* shows inverted test tube for following the gel development

In order to test for the optimum conditions for the preparation of the SF_L_ and SF_C_ gels, corresponding solutions were adjusted at several pH values between 2.5 and 5.0 and kept at 4 °C The gelation time was monitored for about 300 h. The results (Table 1) showed that the SF solutions gelling depended strongly on their pH values. SF_L_ stable gels were achieved at pH values of 3.5 and 4.0 at 192 and 240 h, respectively. However, although solutions of SF_C_ formed stable gels at the same pH values, it consumed much shorter periods corresponding to 48 and 168 at the pH values mentioned above. A lower pH value of 2.5 resulted in an obviously unstable gels for both SF_L_ and SF_C_ solutions. Also, a much higher incubation time was recorded for the stable gels formation at pH higher than tested up to 5 (Fig. 2).

**Table 1:** Gelation times (h) at their corresponding pH values

Gelation time (h)/samples	pH					
	2.5	3.0	3.5	4	4.5	5
SF_L_	168	176	192	240	261	287
SF_C_	40	48	48	168	190	210

### UV–Vis spectroscopy

**Fig. 3:**
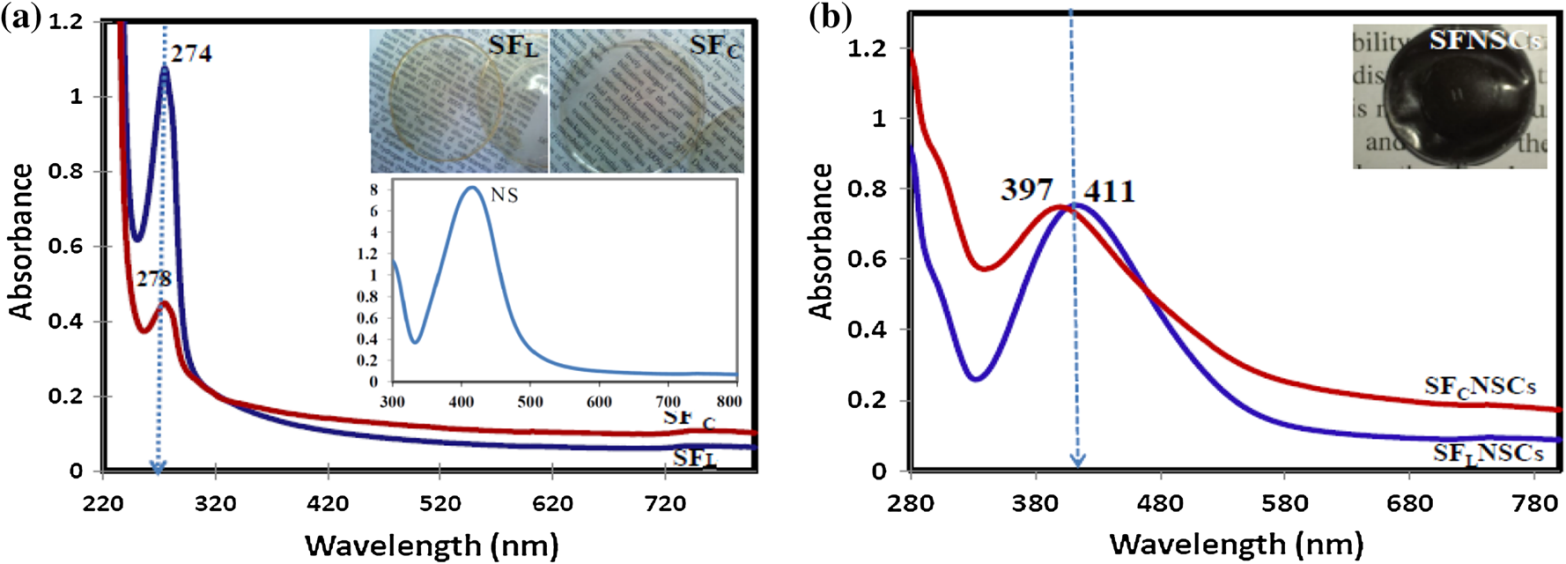
UV–visible absorption spectra for **a** silk fibroin extracted by LiBr (SF_L_), silk fibroin extracted by Ajisawa’s reagent (SF_C_) and nanosilver solution (NS) and **b** composites with nanosilver chitosan (SF_L_NSCs and SF_C_ NSCs). The *inset* pictures show corresponding films in (**a**) and composites (**b**)

Strong bands at 274 and 278 nm characteristic for SF_L_ and SF_C,_ respectively, appeared in Fig. 3a. Chen et al. attributed theses peaks to the π → π* electron transition of the amino acid residues in protein chain of silk fibroin (Autran et al. 2010). Figure 3a also shows that the typical surface plasmon resonance absorption band of NS particles at ~400 nm. The absorption bands of NS in SF_L_NSCs and SFcNSCs solutions were recorded at ~411 and 397 nm, respectively (Fig. 3b).

#### Band gap energy calculations

**Fig. 4:**
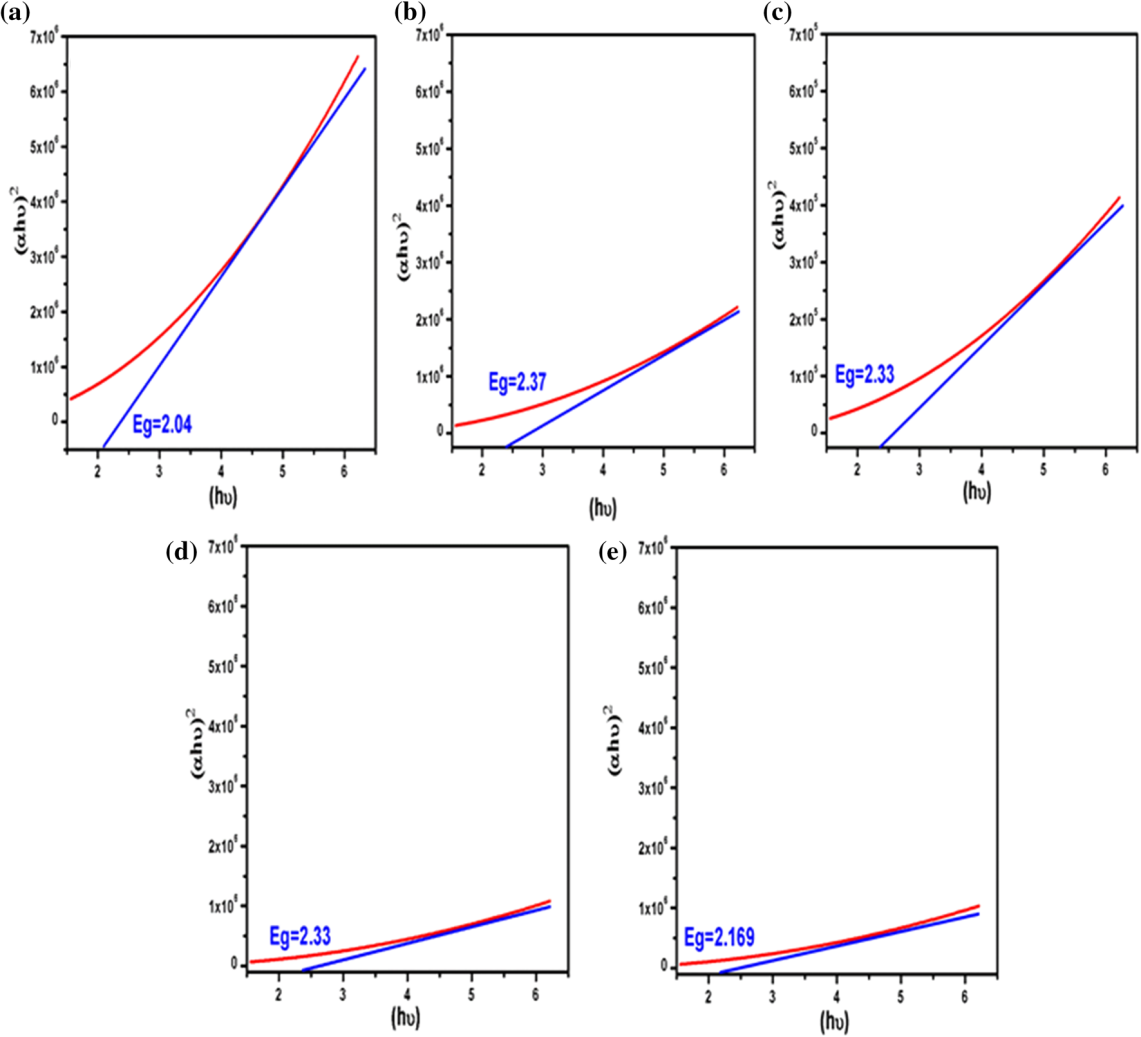
Tacu’s plot of the $(\alpha h\upsilon )^{2}$ as a function of photon energy (*hυ*) of **a** SN, **b** SF_L_, **c** SF_c_, **d** SF_L_NSCs and **e** SF_c_NSCs

The band gap was estimated by Tacu’s relationship:

$$ \alpha = \alpha_{^\circ } (h\upsilon - E_{g  } )^{n} /h\upsilon , $$

where α is absorption coefficient, $h\upsilon$ is the photon energy, $\alpha_{^\circ }$ is constant, *h* is Blank’s constant and *E*_*g*_ is the optical band gap of the material. *n* depends on the type of electronic transition and can be any value between ½ and 3 (Tauc et al. 1966). The energy gaps of the samples have been determined by extrapolating the linear portion of the plots of $(\alpha h\upsilon )^{2}$ against $h\upsilon$ to the energy axis. Figure 4 indicates indirect electronic transition for all samples. The *E*_*g*_ values were calculated to be 2.04, 2.37, 2.33, 2.33 and 2.169 eV for SN, SFL, SFc, SF_L_NSCs and SFcNSCs, respectively. The figure illustrates that the band gap of the composites slightly changed to higher values relative to the silver nanoparticles, which may be due to the polymeric nature of silk fibroin. Moreover, the silver nanoparticles’ incorporation enhances the energy gap values of the pure silk fibroin. The differences have been attributed to variations in the stoichiometric of the synthesis, the impurities content, the crystalline size and the type of electronic transition (Hossain et al. 2008; Zinatloo-Ajabshir and Salavati-Niasari 2015).

### Fourier transform infrared spectroscopy

**Fig. 5:**
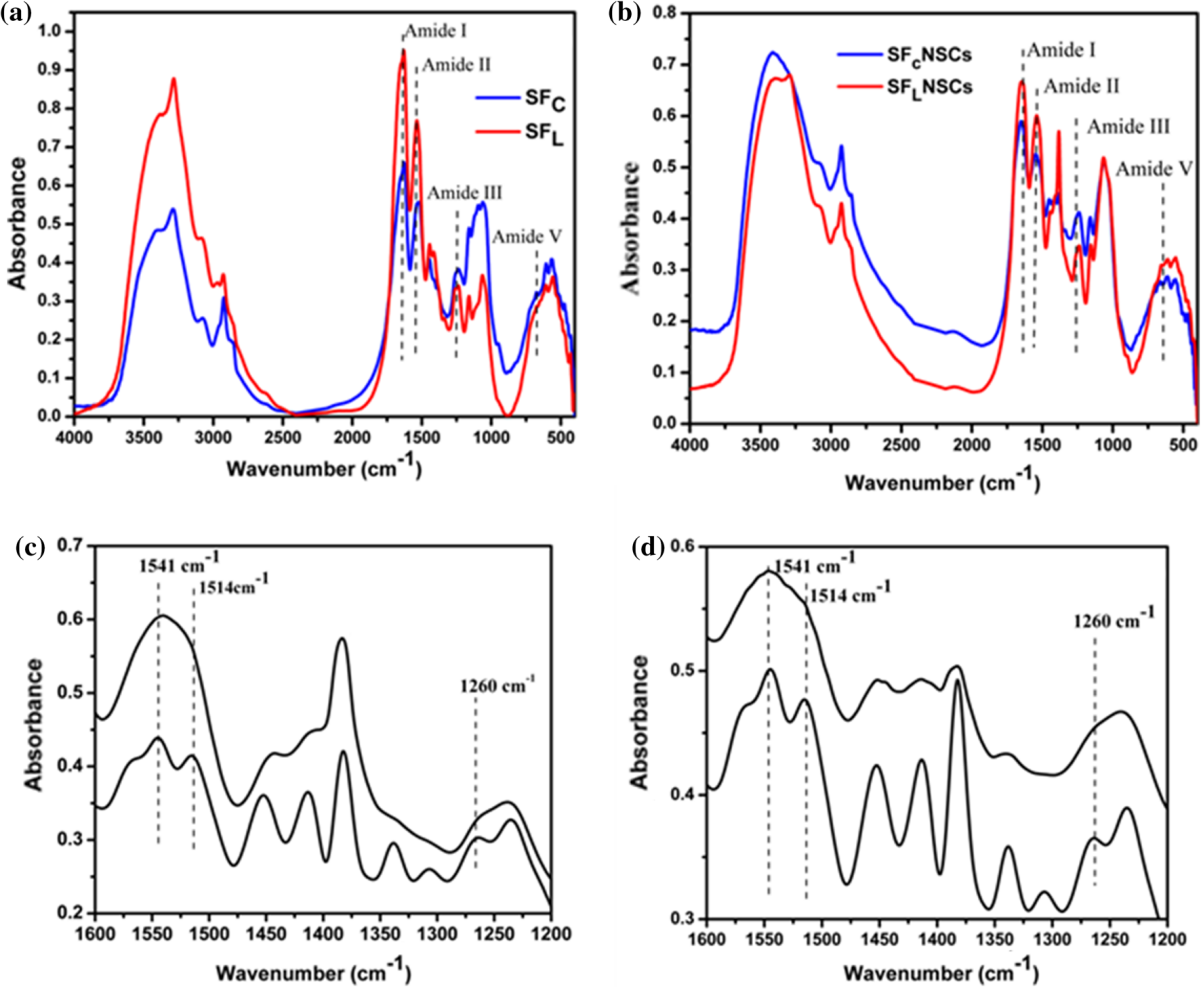
Fourier transform infrared spectra of **a** SF_L_ and SF_C_. **b** FTIR spectra of composites SF_L_NSCs and SF_C_NSCs. Corresponding deconvoluted FTIR Spectra 1200–1600 cm^−1^of **c** SF_C_NSCs and **d** SF_L_NSCs

FTIR spectra of SF_C_ and SF_L_ are shown in Fig. 5a. According to Cristian et al. (2005), the conformations of SF could be a random coil or beta structure and their characteristic bands are amide I, II, III and V modes. These bands are assigned to C=O stretching, NH deformation and O–C–N bending, respectively, while the amide V band arises due to crystallinity. Table 2 shows the assigned bands of Random coil or β fold structure conformation of the silk fibroin and their corresponding composites.

**Table 2:** Comparison of band position shifts for the four amides in the random coil and β structure of SF_C_, SF_L_ and composites SF_L_NSCs, SF_C_NSCs

Samples	Random coil structure	β sheet	SF_L_	SFc	SF_L_NSCs	SF_C_NSCs
Amide I	1650–1660	1625–1640	1633	1633	1655	1655
Amide II	1535–1545	1515–1525	1536	1541	1549	1547
Amide III	1253	1265	1252	1236	1244	1248
Amid V	650	700	609	616	616	606

The absorption bands prove the conformation of the protein is consistent with the absorption of amide I, II, III and V. Therefore, the silk fibroin extracted by either protocol has both α and β structures in their composition. The increased amide I and II intensities in SF_L_ indicated more water-soluble protein contents, while β sheet assigned bands appeared more intense at SF_C_ spectrum proving its higher crystallinity. The composites SF_L_NSCs and SF_C_NSCs exhibited shifts in the absorption bands of SF proving interaction between NS and NSCs (Fig. 5b).

### X-ray diffraction patterns (XRD)

**Fig. 6:**
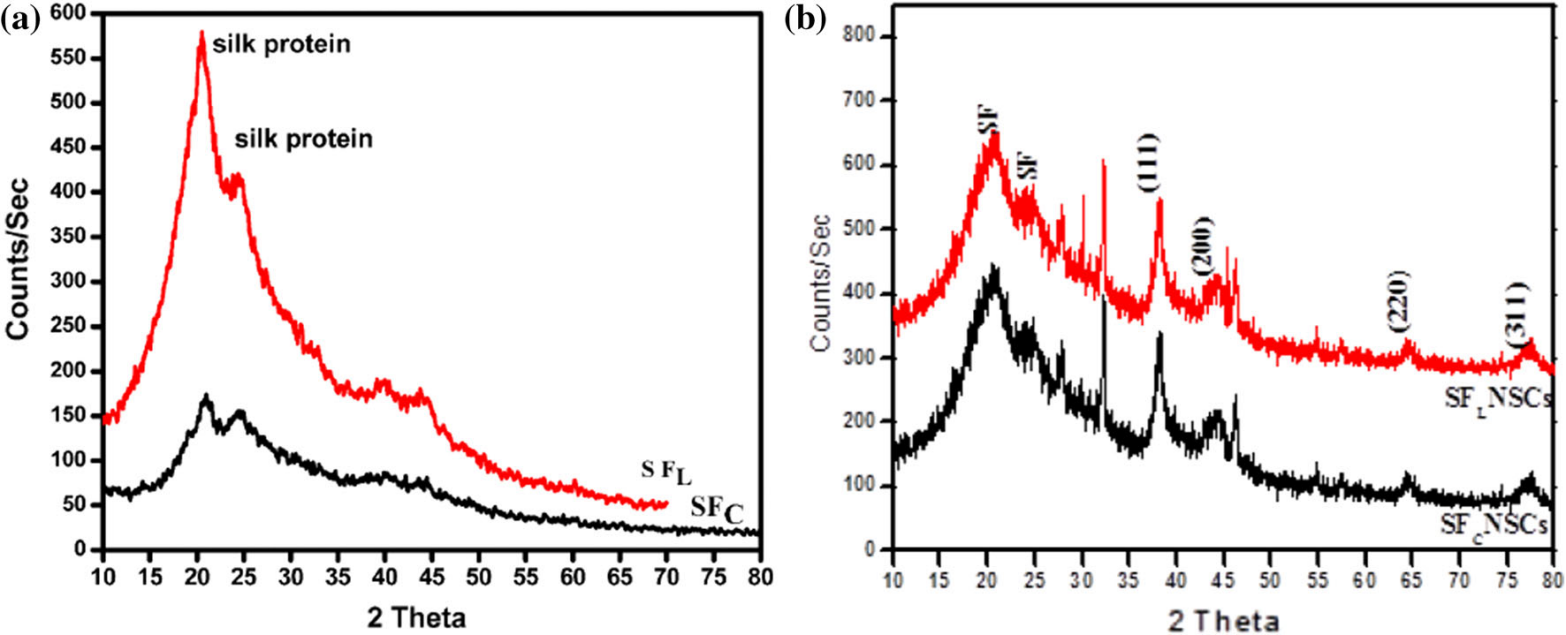
XRD patterns of **a** SF_L_ and SF_C_ and **b** characteristic Ag bands embedded in the silk composites recorded at 2*θ* of 38.09°, 43.92°, 64.29° and 77.51° corresponding to 111, 200, 220 and 311 planes for each of SF_L_NSCs and SF_C_NSCs

XRD spectra of SF_L_ and SF_C_ are shown in Fig. 6a. Silk fibroin protein peaks arising at 2*θ* = 20° and 25° are attributed to the β-sheet crystalline structure of fibroin (Andiappan et al. 2013). The XRD peaks of SF_C_ indicate more crystalline structure compared to those of SF_L_ confirming and therefore, FTIR results.

For the composites SFNSCS (Fig. 6b), in addition to silk fibroin peaks, XRD pattern revealed four peaks corresponding to 4 diffraction facets of silver recorded at 2*θ* = 38.09°, 43.92°, 64.29° and 77.51°. The discernible peaks can be indexed to the planes (111), (200), (220) and (311), respectively, corresponding to face-centered cubic structure of silver according to JCPDS (No. 4-0783) which confirms the binding of NSCs to silk fibroin. A few intense additional and yet unassigned peaks were also noticed in the vicinity of the silver characteristic peaks. These sharp Bragg peaks might have resulted from some bioorganic compounds/protein(s) present in the composite (Pavani et al. 2013).

### Transmission electron microscope (TEM)

**Fig. 7:**
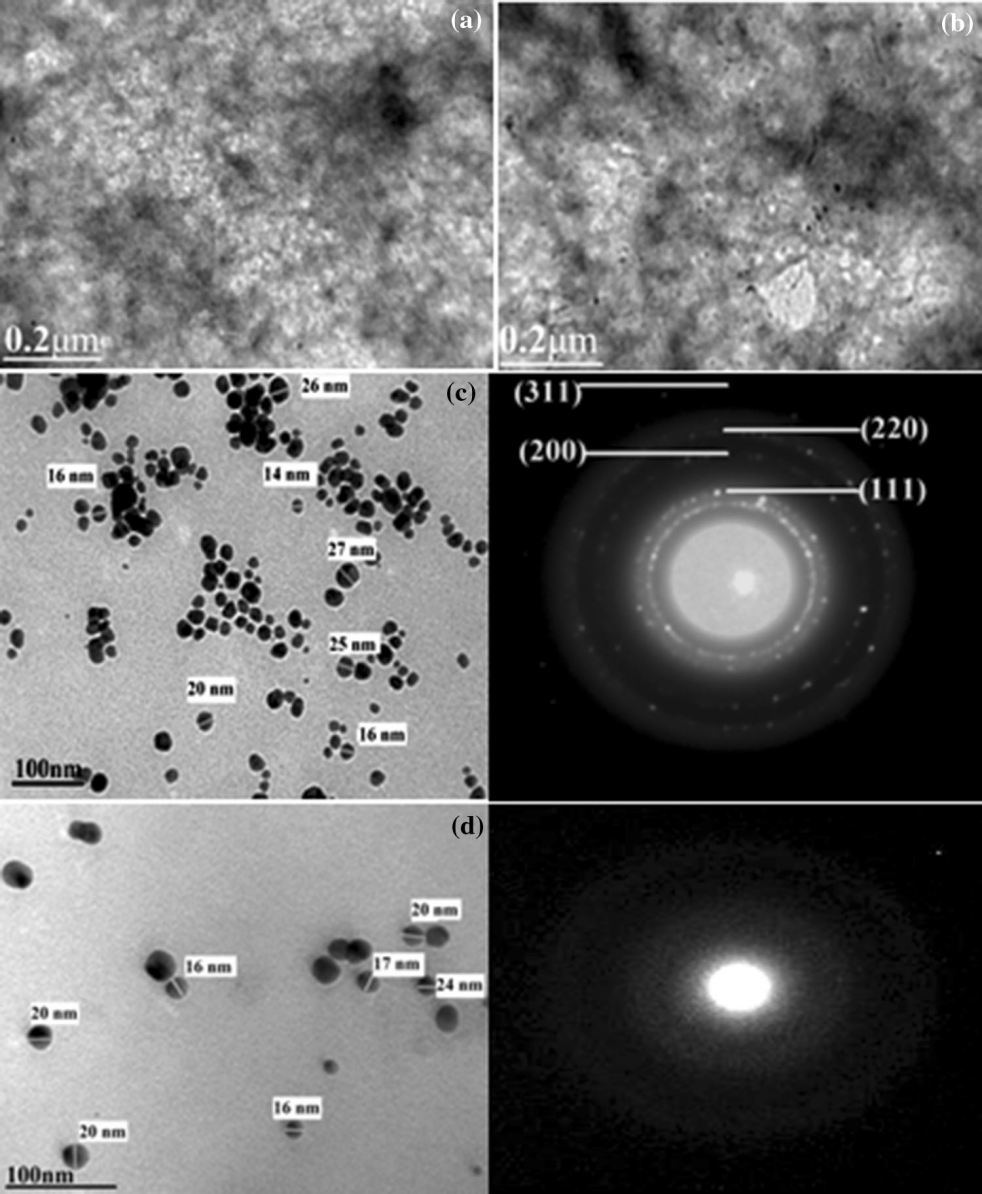
TEM micrographs showing the structure of silk fibroin **a** SF_C_, **b** SF_L_, composites **c** SF_c_NSCs and **d** SF_L_NSCs and corresponding SAED patterns

Figure 7a shows the partially definite structure for silk fibroin extracted using the two protocols. After impregnation with chitosan silver nanoparticles, SF_L_NSC_S_ and SF_C_NSC_S_ reveal crystalline features. The selected area electron diffraction (SAED) patterns obtained for a representative composite have face-centered cubic (fcc) crystallographic structure. A number of well-dispersed nanoparticles which are spherical in shape and separated from each other with higher crystallinity are recorded for the SF_C_NSCs composite which showed well-defined diffraction rings.

### Antimicrobial activity assessment

**Fig. 8:**
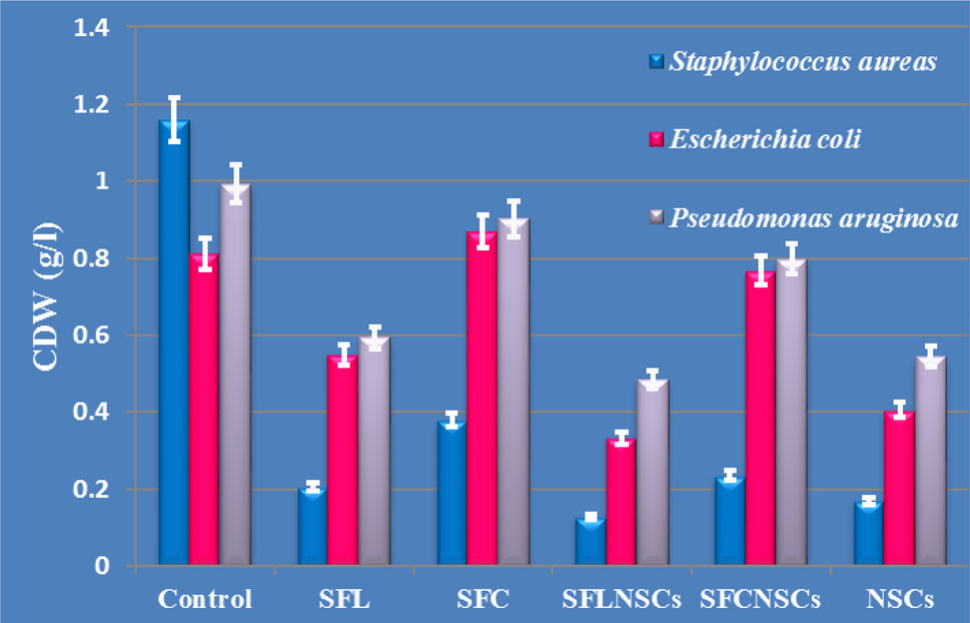
Antibacterial activities of the tested films SF_L_ and SF_C_ and their composites SF_L_NSCs, SFcNSCs against several types of gram +ve and gram −ve medically relevant bacteria demonstrating highest bactericidal activity for SF_L_ and it’s composite

The results represented in Fig. 8 showed that all the tested pellicles had a more acute effect on the gram-positive *S. aureus* cells compared to the gram-negative ones of *E. coli* and *P. aeroginosa*. This could be relied on the morphological and structural differences of the tested bacteria (Abdel-Fattah et al. 2014).

The histogram also shows that the films, developed using silk fibroin extracted by LiBr (SF_L_), had a more intense antibiotic activity than those made using silk fibroin processed with Ajisawa’s reagent. The former resulted in about 95 % growth reduction while the latter resulted in only 67 %. This was probably due to the longer time necessary to completely dissolve the protein using the second protocol thus exposing the protein to a high temperature of 60 °C as well as mechanical stirring for a longer time, consequently, causing structural changes.

**Fig. 9:**
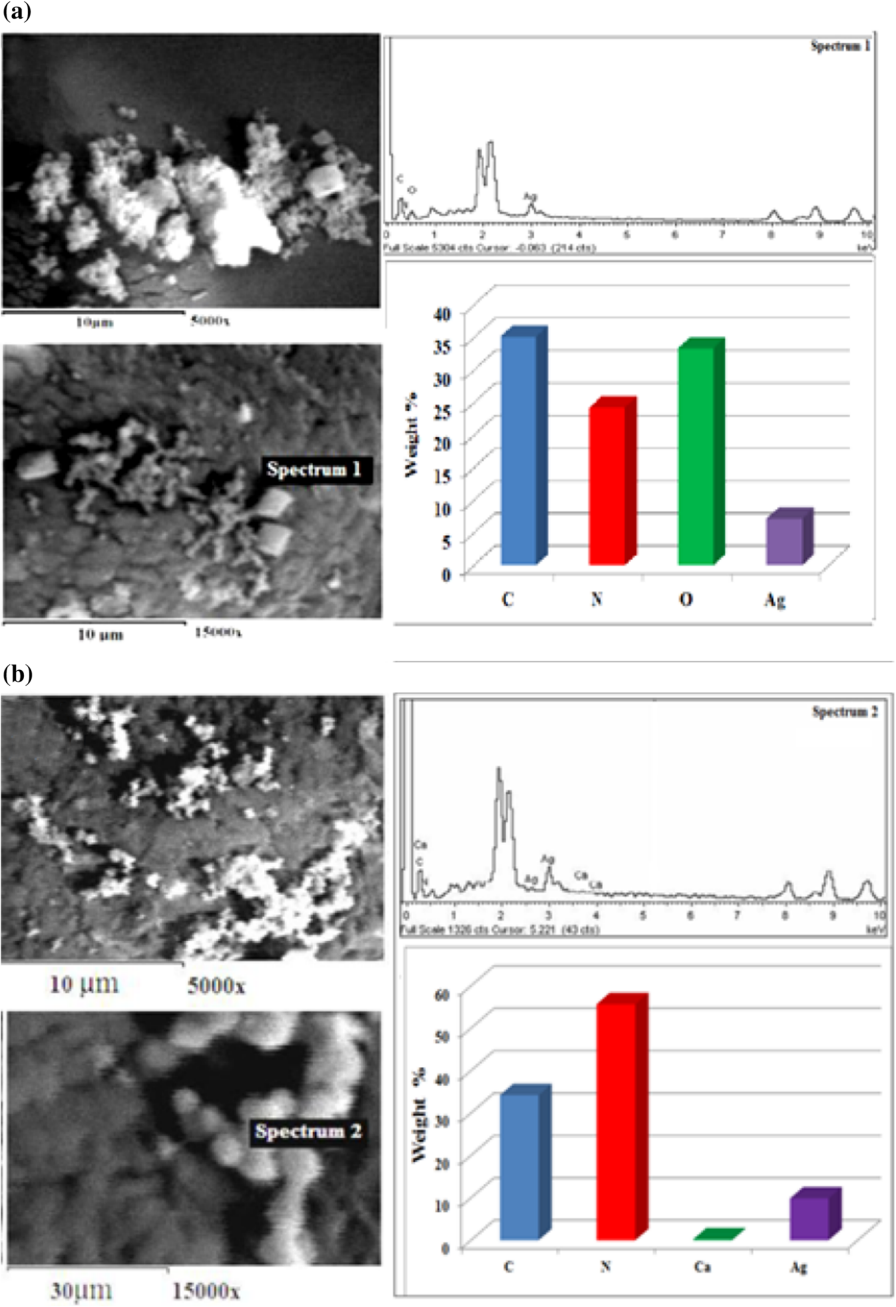
SEM of the composite films with their EDAX on the selected area of **a** SF_L_NSCs and **b** SF_C_NSCs after subjecting films to *S. aureus*, proving the massive destruction of bacterial cell walls especially for **a** SF_L_NSCs composite with less nitrogen and silver contents

The massive destruction of the tested bacterial cells is clearly elucidated through SEM examination (Fig. 9a). Comparing the remaining percentages of silver nanoparticles, after the exposure of the SF_L_NSCs and SF_C_NSCs composites to the bacteria, proves that more silver nanoparticles were involved in the bacterial invasion when SF_L_NSCs composites were used. Therefore, its higher antibacterial results are proved among all the tested pellicles.

## Discussion

The present results proved that the decrease in the pH of the SF solutions favors the conformation transition from silk I to silk II which is related to the β-sheet formation. This is in agreement with the results of Nagarkar (2010) and could be explained by the fact that lowering the pH leads to a reduction in the negative charges due to the protonation of the amino acids. The later may promote protein refolding to a more ordered state stabilized by the hydrogen bonding between the chains and accompanied by an exclusion of water (Nagarkar 2010). The corresponding shorter gelation time of SFc (silk fibroin extracted by Ajisawa’s reagent) could be explained through considering its structure. FTIR results revealed more insoluble beta sheet in it compared to SF_L_ (silk fibroin extracted by LiBr) having more alpha helix. The β-sheet is comparatively more ordered and favors shorter gelation time.

The red shift of amide I absorption band of both composites from 1633 to around 1655 cm^−1^ Fig. 5a, indicates blending with NSCs which induce further changes in SF protein structure. The peak at 1387 cm^−1^ recorded for composites SF_C_NSCs and SF_L_NSCs showed increased intensity for the first one indicating more binding of NSCs to silk fibroin inconsistency with the work of Ahmad et al. (2011).

Figure 5b also shows that the stretching frequency C=O band exhibited a blue shift in comparison to that of the free silk, denoting chelation. This shift was due to the reduction of the double bond character of the oxygen to the metal center in agreement with results reported (Reza and Morsali 2011).

For a better comprehension of these results, a deconvolution of amide regions was performed, (Fig. 5). The amide I peak split into two small ones at 1650 and 1622 cm^−1^ where the last one indicates further increased β sheet structure as a result of interaction between chitosan and silk fibroin. The amide II band is divided into two peaks at 1514 and 1541 cm^−1^ confirming, therefore, that the composite contains both random coil and β sheet structures. The new peak appearing at 1260 cm^−1^ presented conformation transition from random coil to β-sheet of SF (Luangbudnark et al. 2012). These results lead to the fact that in addition to the chitosan role in protecting silver nanoparticles from agglomeration by enveloping them, it can also increase the transition from random coil to beta sheet of SF.

The crystallite domain size was calculated from the width of the XRD peaks, assuming that they are free from non-uniform strains, using the Scherrer formula (Cullity 1978).

$$ D = \frac{0.9 \lambda }{{\beta { \cos }\theta }}, $$

where *D* is the average crystallite domain size perpendicular to the reflecting planes, *λ* is the X-ray wavelength (1.5418 Å), *β* is the full width at half maximum (FWHM) and *θ* is the diffraction angle. Similar applications of Scherrer formula were used for assessing crystallite size domain for composites and nanoparticles (Salavati-Niasari et al. 2008, 2009; Zinatloo-Ajabshir et al. 2015; Zinatloo-Ajabshir and Salavati-Niasari 2014). The intensity of the Bragg reflections suggests strong X-ray scattering centers in the crystalline phase. It could possibly result from metalloproteins in XRD spectra of both SF_C_NSCs and SF_L_NSC structures with an average particle size of 13–19 nm, respectively, with cubic shape. The obtained results denote that the incorporation of silver nanoparticles into the silk matrix does not affect its crystallite size.

The bactericidal effect shown in the histogram reveals that the SF_L_ and SF_C_ films, developed using silk fibroin extracts as a sole constituent, resulted in an obvious growth reduction of the tested strains, proving, therefore, that the silk fibroin itself has an effective antibacterial activity. These results have a logical explanation as the silk fibroin is the main constituent of silkworm cocoons which is initially meant to protect the pupae stage of silk worm in nature for a certain period of time. It would not be surprising that a kind of protection against bacteria is present in the fibers; otherwise, they would be biodegraded very fast and would not be able to protect the pupae till their complete development into mature butterflies and continue the life cycle of silkworm.

A comprehensive scientific explanation could be attributed to Pelegrini et al. (2008). The latter, in order to develop a novel approach to control common bacterial infections in plants, identified a number of defense peptides with antibacterial activities. Their characterization, using the amino acid sequencing method, clearly revealed that the glycine-rich protein was responsible for such activity. The mode of action of this protein seems to be via its interaction with the lipid layer of the bacterial cell wall surface leading to membrane permeability and consequent cell death (Pelegrini et al. 2008). On the other hand, many reports studied the structure of silk fibroin originating from the silkworm (*B. mori*). These studies showed that it is an insoluble protein formed of layers of antiparallel beta sheets. Although it is composed of 18 different amino acids, it has a high glycine concentration protein as it contains approximately 43 % of its molecular weight glycine which allows for tight packing of the sheets and, therefore, contributes to the silk’s rigid structure and tensile strength. Therefore, we suggested that, similarly, the antibiotic activities of silk fibroin against the tested microorganisms could also be attributed to the presence of the high concentration of glycine in silk fibroin protein. This assumption was previously confirmed by the study of Ponnuvel and Yamakawa (2002). The latter found that the presence of such concentration of glycine in the silk fibroin extracted from silkworm was, among others, one of the main causes of its antimicrobial ability.

Moreover, the results in Fig. 5, illustrating the FTIR spectra of the dissolved silk fibroin, clearly elucidated the characteristic peaks for the random coil and α-helix conformation (water-soluble fraction of the protein) as well as the β-sheet conformation (water-insoluble fraction of the protein) at the wavenumber of 1650 and 1235 cm^−1^, respectively (Wang et al. 2004; Huang et al. 2011). The present data showed that although both protein states coexist together in the silk fibroin samples obtained using either protocol, the protein in α-helix was more marked when the LiBr reagent was used. TEM results additionally revealed that the SFcNSCs is more crystalline in nature than SF_L_NSCs. However, several studies showed that crystallization is directly dependent on the presence of more β sheet structure. Therefore, the higher antibiotic activity of SF_L_ films is explained, since only proteins in the soluble state could attack and penetrate the cell membranes of the tested microorganisms more efficiently.

**Fig. 10:**
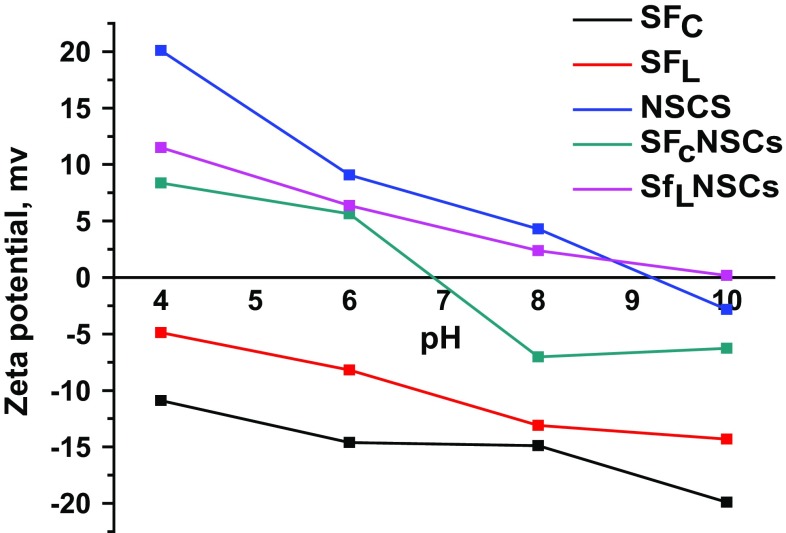
Zeta potential values at several pH of all the prepared silk fibroin films SFc and SF_L_, silver/chitosan (Ag6d3), and composites (SFcNSCs) and (SF_L_NSCs)

The Zeta potential results (Fig. 10) revealed that, the NSCs possess a positive value with maximum of 20 mv indicating partially stable nanoparticles, while pure SF possesses a negative zeta potential values ranging between −4.8 and −19.9 mv. On the contrary, SF recorded positive values after its incorporation with NSCs. These results give more explanation for its higher antibacterial activity via electrostatic attraction between the negatively charged cell membranes of the bacteria and the positively charged composites. Therefore, Zeta potential is an essential parameter for the characterization of the stability of SF and SFNSCs suspensions.

## Conclusion

Silk fibroin proved to be more readily dissolved in LiBr solution than in Ajisawa’s reagent. Although both protocols eventually produced highly crystalline composites, SF_C_ exhibited higher crystallinity with shorter gelation time. The optical energy calculated from all constructs indicated indirect electronic transitions. The regenerated silk fibroin exhibited antibacterial activity against the tested gram-positive and gram-negative bacteria. Moreover, reduction ability for the bacterial growth by SNCs composite was greatly enhanced by the inclusion of silk fibroin especially upon extraction with LiBr solvent system. The turbidity measurements showed that the bacterial inhibition increased up to 82 % due to the combined effects of SN, Cs and SF. NSCs film has intermediate effect between both composites with that of Li imparting the highest effect. Such developed membranes could, therefore, be prospectively used as a commercial product for wound healing treatment. The SFNSCs composite advantages are 1) natural, having antibacterial activities. 2) Expected to be non-immunogenic and could be processed easily into several forms. 3) Reproducible and could be directly applied in several biomedical applications. However, the darkness of the developed constructs could be controlled by optimizing the silver nanoparticles’ concentration within the construct in a future work which will need further relevant bactericidal assessment too.
